# Supplementary figures and images for: Ultrafast endocytosis in mouse cortical inhibitory synapses
Source: bioRxiv. 2025 Jun 8:2025.06.06.658279. Preprint. [Version 1] doi: 10.1101/2025.06.06.658279 (PMC12157488; doi:10.1101/2025.06.06.658279)

Figure S1. Eddings, et al.

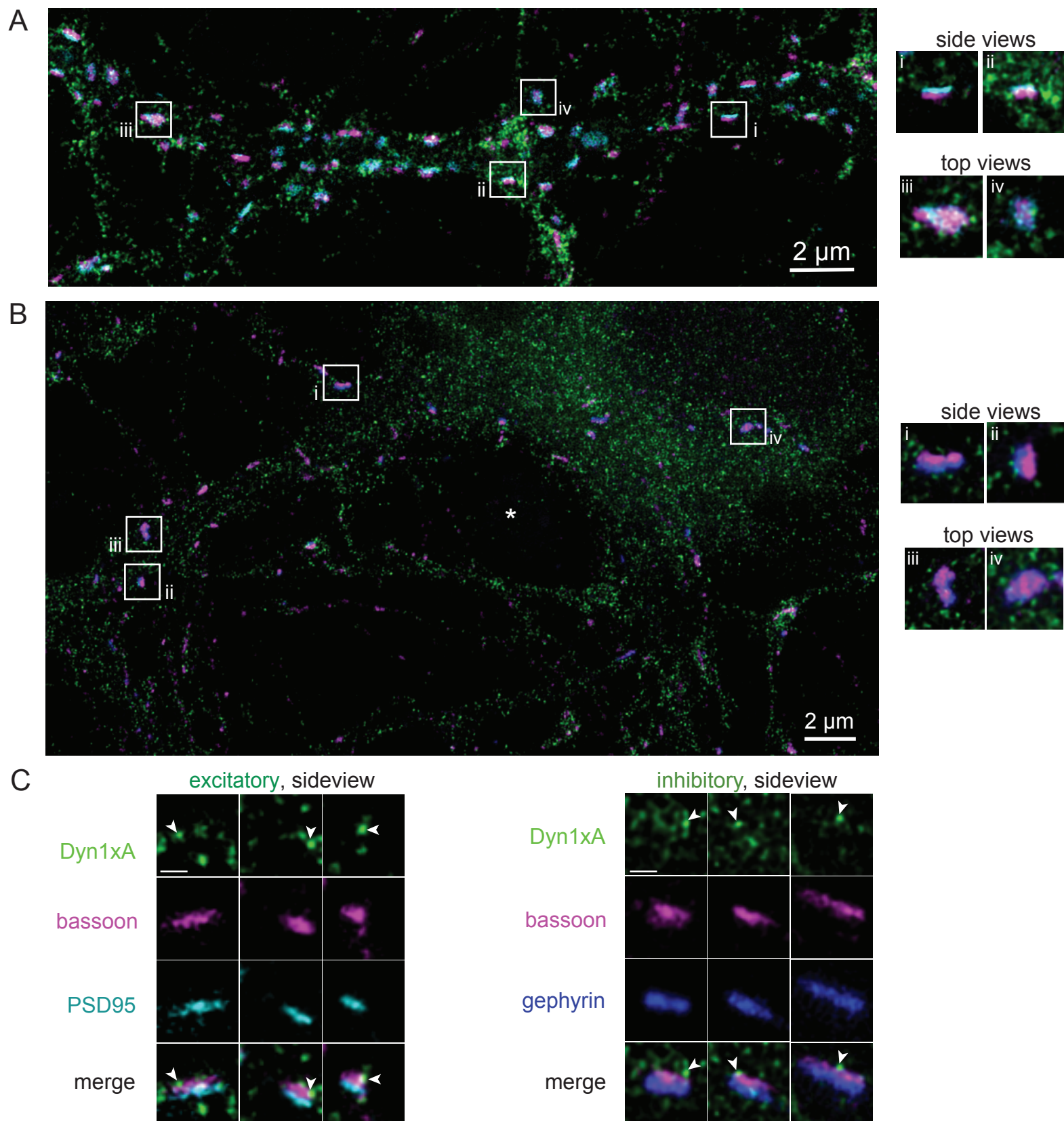

Figure S2. Eddings, et al.

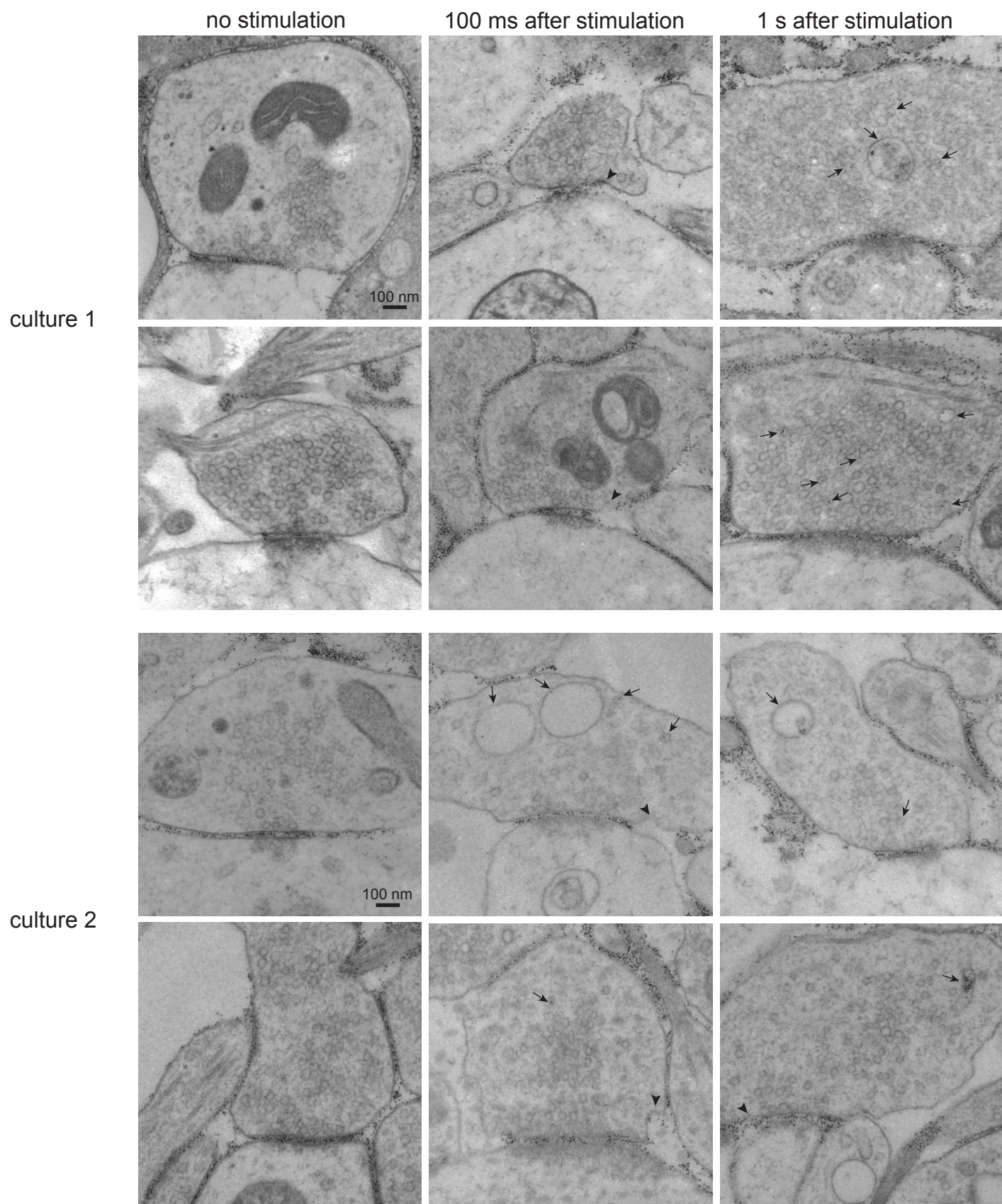

Figure S3. Eddings, et al.

A

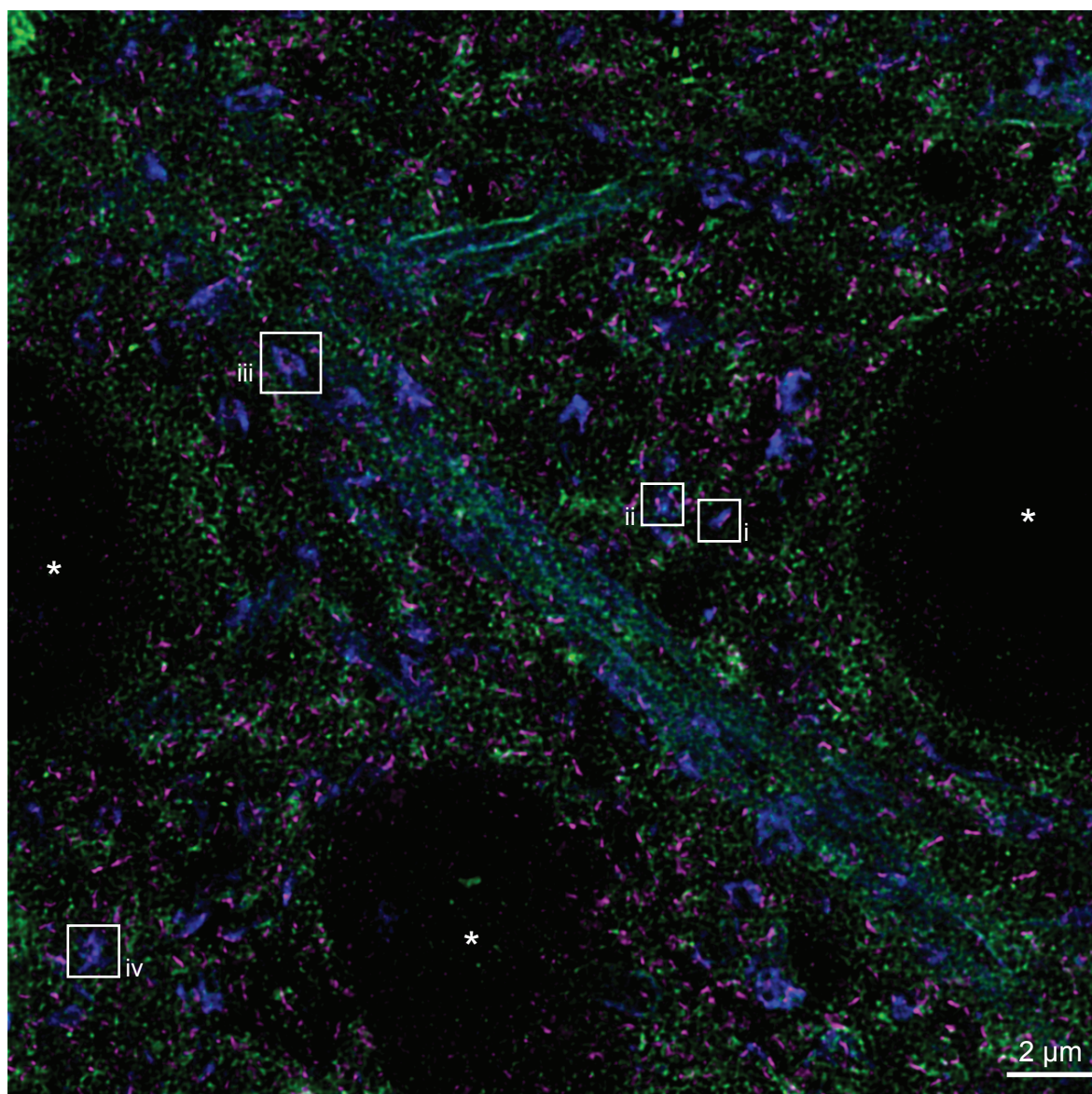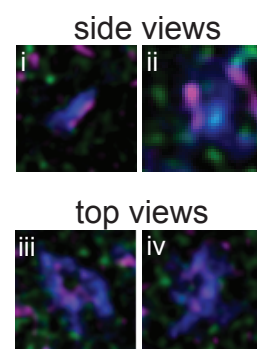

B

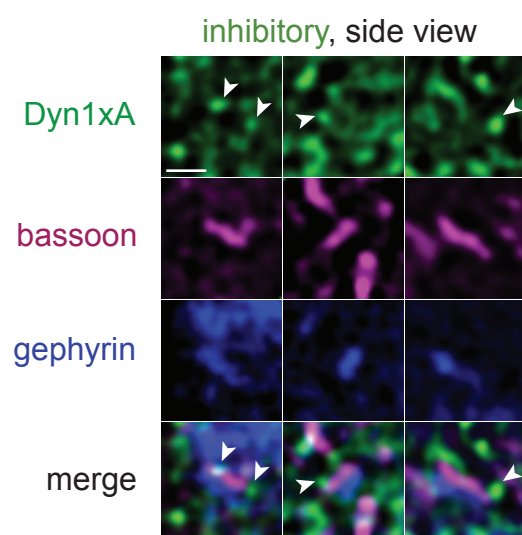

Figure S4. Eddings, et al.

A

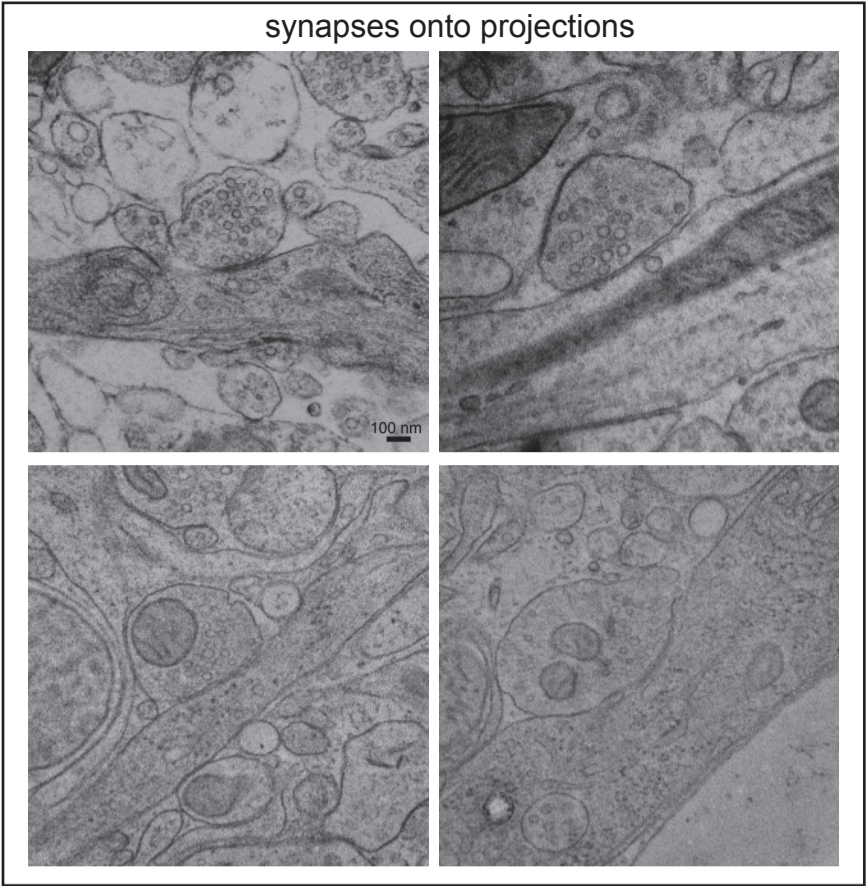

B

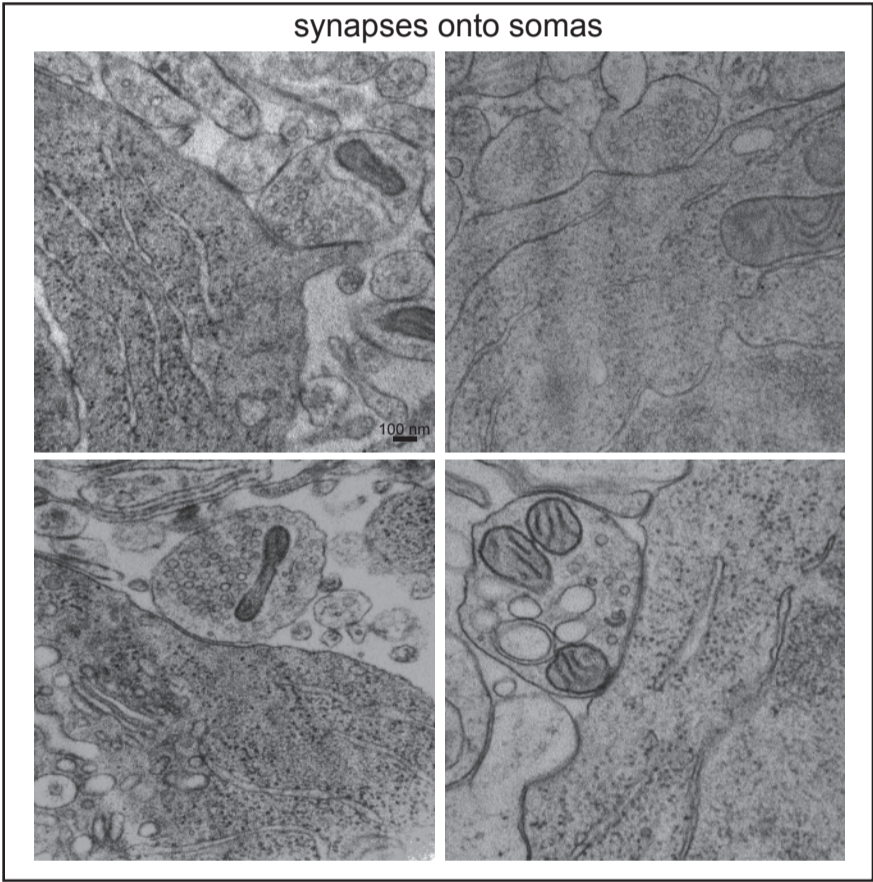

C

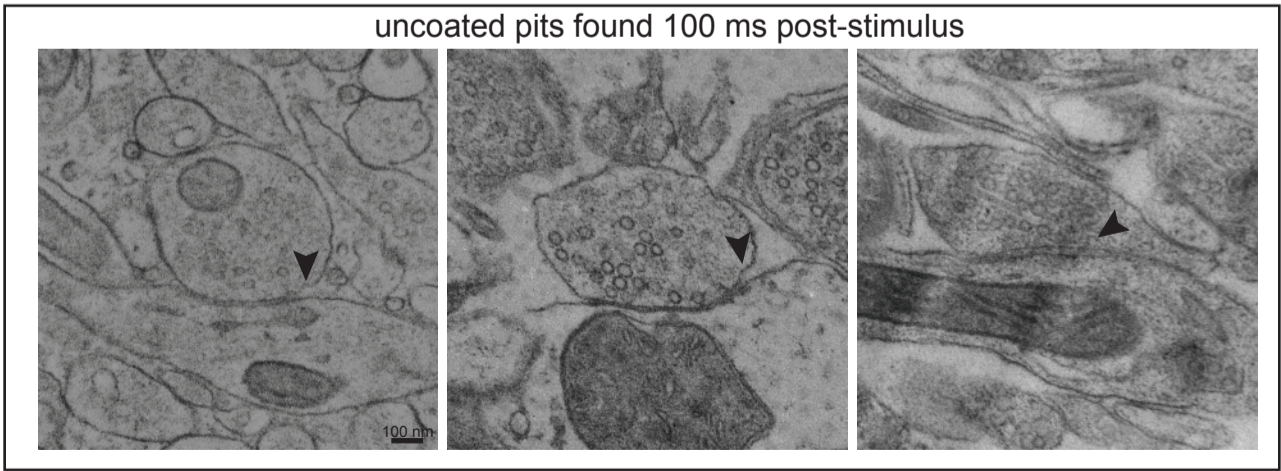

Supplement: Supplement 1 — Figure S1. Additional STED images for Figure 1. Overview 2D, three-color STED images of cultured mouse cortical synapses. Example side view (i and ii) and top view (iii and iv) synapses are highlighted as panels. (A) Excitatory synapses observed on projections. (B) Inhibitory synapses observed on a cell body (nucleus devoid of staining is denoted by * symbol). (C) Example Dyn1xA puncta in side view excitatory and inhibitory synapse images, which were present but not analyzed. Scale bars: 300 nm unless noted. Figure S2. Additional EM images for Figure 2. Example electron micrographs of cultured mouse cortical neurons that have undergone zap-and-freeze at the indicated time points. Uncoated endocytic pits (black arrowheads) and ferritin-positive membrane structures (black arrows) are indicated. Here no distinction was made between excitatory or inhibitory synapses. Scale bars: 100 nm. Figure S3. Additional STED images for Figure 3. (A) Overview 2D, three-color STED image of a cortical region in an acute mouse brain slice. Example side view (i and ii) and top view (iii and iv) synapses are highlighted as panels. Nuclei devoid of staining are denoted by * symbols. (B) Example Dyn1xA puncta in side view inhibitory synapse images, which were present but not analyzed. Scale bar: 300 nm unless noted. Figure S4. Additional EM images for Figure 4. Example electron micrographs of acute mouse brain slices that have undergone zap-and-freeze. Synapses shown with non-prominent postsynaptic densities onto either (A) projection shafts or (B) somas/cell bodies. (C) More example uncoated pits (black arrowheads) found 100 ms post-stimulus in synapses with non-prominent postsynaptic densities. Scale bars: 100 nm. [file media-1.pdf]
